# Poor sleep versus exercise: A duel to decide whether pain resolves or persists after injury

**DOI:** 10.1016/j.bbih.2023.100714

**Published:** 2023-12-12

**Authors:** David M. Klyne, Brendan A. Hilliard, Michele Y. Harris, Mamta Amin, Michelle Hall, Manuela Besomi, Sanam Mustafa, Scott F. Farrell, Oliver Rawashdeh, Felicity Y. Han, Paul W. Hodges, Nagat Frara, Mary F. Barbe

**Affiliations:** a NHMRC Centre of Clinical Research Excellence in Spinal Pain, Injury and Health, School of Health and Rehabilitation Sciences, The University of Queensland, Brisbane, 4072, Australia; b Aging + Cardiovascular Discovery Center, Lewis Katz School of Medicine of Temple University, Philadelphia, 19140, USA; c Centre for Health, Exercise and Sports Medicine, School of Health Sciences, The University of Melbourne, Melbourne, 3010, Australia; d School of Biomedicine, The University of Adelaide, Adelaide, 5005, Australia; e RECOVER Injury Research Centre, NHMRC Centre of Research Excellence in Better Health Outcomes for Compensable Injury, The University of Queensland, Brisbane, 4029, Australia; f School of Biomedical Sciences, The University of Queensland, Brisbane, 4072, Australia; g Australian Institute for Bioengineering and Nanotechnology, The University of Queensland, Brisbane, 4072, Australia

**Keywords:** Pain, Transition to chronicity, Poor sleep, Exercise

## Abstract

Poor sleep is thought to enhance pain via increasing peripheral and/or central sensitization. Aerobic exercise, conversely, relives pain via reducing sensitization, among other mechanisms. This raises two clinical questions: (1) does poor sleep contribute to the transition from acute-to-persistent pain, and (2) can exercise protect against this transition? This study tested these questions and explored underlying mechanisms in a controlled injury model. Twenty-nine adult female Sprague-Dawley rats performed an intensive lever-pulling task for 4 weeks to induce symptoms consistent with clinical acute-onset overuse injury. Rats were then divided into three groups and exposed for 4 weeks to either: voluntary exercise via access to a running wheel, sleep disturbance, or both. Pain-related behaviours (forepaw mechanical sensitivity, reflexive grip strength), systemic levels of brain derived neurotrophic factor (BDNF), estradiol and corticosterone, and white blood cells (WBC) were assessed pre-injury, post-injury and post-intervention. Mechanical sensitivity increased post-injury and remained elevated with sleep disturbance alone, but decreased to pre-injury levels with exercise both with and without sleep disturbance. Reflexive grip strength decreased post-injury but recovered post-intervention—more with exercise than sleep disturbance. BDNF increased with sleep disturbance alone, remained at pre-injury levels with exercise regardless of sleep, and correlated with mechanical sensitivity. WBCs and estradiol increased with exercise alone and together with sleep disturbance, respectively. Corticosterone was not impacted by injury/intervention. Findings provide preliminary evidence for a role of poor sleep in the transition from acute-to-persistent pain, and the potential for aerobic exercise to counter these effects. BDNF might have a role in these relationships.

## Highlights

• First evidence of a role for poor sleep in the transition from acute to persistent pain.
• Aerobic exercise protects against the pain-inducing effects of poor sleep.
• Brain-derived neurotrophic factor might have a role in these relationships.

## 1 Introduction

Poor sleep frequently co-occurs with acute injury (Klyne et al., 2017), a painful flare-up (Costa et al., 2021), or ongoing, chronic pain (Smith et al., 2004). Contrary to the traditional view that pain interferes with sleep, emerging work suggests the opposite is also true—poor sleep increases pain (Babiloni et al., 2020; Farrell et al., 2023). An intriguing question is whether poor sleep contributes to the transition from acute to chronic pain. Until now, no adequately controlled studies have quantitatively answered this.

Mechanisms underlying the relationship between sleep and pain remain to be fully elucidated. One possible explanation involves the capacity for poor sleep to drive mechanisms that sensitize pain pathways (Chang et al., 2022). Changes in sleep patterns alter circadian rhythms and expression levels of neurotrophic factors such as brain-derived neurotrophic factor (BDNF) (Cain et al., 2017; Schmitt et al., 2016), which serves as a key regulator of synaptic plasticity in the peripheral and central nervous system (Nijs et al., 2015). Repeated or persistent periods of sleep loss/poor sleep quality could lead to sustained increases in circulating BDNF that drive central sensitization and maladaptive neuroplasticity, which, in turn, drive the transition from acute to chronic pain.

Conversely, aerobic exercise is associated with reduced pain sensitization (Tan et al., 2022). This might underpin why aerobic exercise is one of the most effective treatments for pain (Geneen et al., 2017). It also raises the interesting question of whether exercise protects against the pain-inducing effects of poor sleep. Confirmation of this would have important clinical implications.

This study explored these knowledge gaps by longitudinally profiling mechanical sensitivity, reflexive grip strength (linked to myalgia (Hadrevi et al., 2019)), and systemic biomarkers associated with pain sensitization in rats exposed for 4 weeks to either sleep disturbance, aerobic exercise, or both, after acute-onset overuse injury. To circumvent the confounding impact of acute stress (on the immune system and pain) inherent in current models of poor sleep and forced exercise, we applied a method with greater translational relevance to induce intermittent disturbed sleep and provided aerobic exercise in the form of “voluntary” access to a running wheel. We hypothesised that post-injury pain sensitivity would persist and/or increase with sleep disturbance, but that exercise would offset these effects and be associated with changes in BDNF.

## 2 Materials and methods

### 2.1 Overall study design

**Fig. 1:**
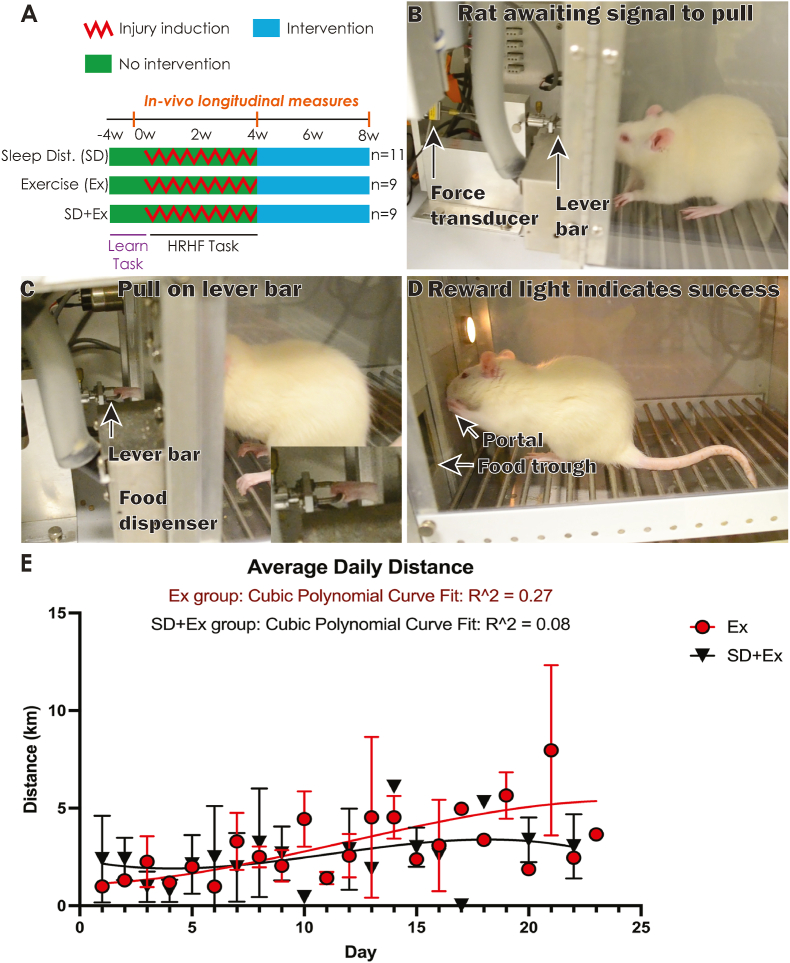
Study design, injury model, and daily running distance. (A) Study design. n = number of animals per group; SD = sleep disturbance; Ex = whole body aerobic exercise via running wheel access; SD+Ex = sleep disturbance and exercise combined. (B–D) Images showing the overuse injury model. Rats learned and then performed a high-repetition high-force (HRHF) reaching and lever pulling task (four 30-min sessions/day, 3 days/week, for 4 weeks). (E) Average daily running distance for Ex and SD+Ex rats.

Full descriptions of materials and methods are in Appendix A. Fig. 1A depicts the experimental design. Twenty-nine adult female Sprague-Dawley rats performed an intensive lever-pulling task (Fig. 1B–D) for 4 weeks to induce symptoms consistent with clinical acute-onset overuse injury in forearm neuro-musculoskeletal tissues (Barbe et al., 2013). Rats were then randomly allocated into 3 groups and exposed for 4 weeks to either: voluntary aerobic exercise via access to a running wheel (Ex; n = 9), sleep disturbance (SD; n = 11), or both (SD+Ex; n = 9). Longitudinal measures of pain-related behaviours and systemic biomarkers of neuroimmune activity were obtained: (1) pre-injury, (2) immediately following 4 weeks of lever-pulling (post-injury), and (3) immediately after the 4-week intervention phase (post-intervention). All rats were handled at least 15 min/day and provided with tunnels and chew toys in home cages. Experiments were approved by Temple University’s Institutional Animal Care and Use Committee and were compliant with NIH guidelines for the use of laboratory animals.

### 2.2 Acute-onset overuse injury by an intensive lever-pulling task

Rats trained for 4 weeks (15 min/day, 5 days/week) to learn a high force lever-pulling task, ramping up from naïve to high loads, as described (Barbe et al., 2013). Rats then went on to perform a high-repetition high-force reaching and lever‐pulling task (2 h/day, four 30-min sessions/day; 3 days/week) for 4 weeks in a customized apparatus (Fig. 1B–D; see Appendix A for details).

### 2.3 Sleep disturbance

Sleep was disturbed by frequently arousing the rats during the light-phase for 12 h/day (0600–1800, as rats are nocturnal) on four random days/week to avoid circadian and sleep-pattern adaptations. This was achieved by replacing an old object with a new stimulatory object in their cage when the rat appeared drowsy. Stimulatory objects included plastic toys and tubes of different sizes, tube lids, marbles, and nesting material. When necessary, additional arousal was achieved by disturbing the bedding or introducing objects containing sucrose food reward pellets that could be sensed but not accessed by the animal. Cages were also exchanged twice/day (1200 and 1400) for fresh ones to provide additional stimulation. This method avoids the confounding impact of acute stress associated with current sleep restriction/disturbance methods and better reflects “real-world” poor sleep.

### 2.4 Aerobic exercise

Rats were given the opportunity to perform whole body aerobic exercise in the form of “voluntary” access to a running wheel. This was provided via free access to a built-in running wheel (with an automated rotameter for measuring distance) from within their cage for 12 h during the dark phase (1800-600), 5 days/week, for 4 weeks. Rats were housed separately during this time to allow unrestricted access to the wheel. Wheel access was not permitted between 600 and 1800 (light-phase) to avoid the stimulatory effects of the task that could alter normal sleep patterns. Rats were returned to their usual cage (i.e., without a wheel) during the light-phase. Remaining rats were housed in their usual cage without wheels. All were provided tunnels and chew toys in their home cages and were handled daily, which allowed for some voluntary physical activity.

### 2.5 Measures of pain and discomfort

Forepaw sensitivity to mechanical stimulation was assessed bilaterally using von Frey monofilaments (North Coast Medical, Inc. CA, USA), as described (Clark et al., 2004). The number of forelimb withdrawal responses out of 10 probings was quantified separately for four monofilament sizes: 0.4, 1, 4, and 8 g-force (presented hereafter as centiNewtons, cN). Reflexive grip strength was tested using a rat grip strength meter (1027SR‐D58, Columbus Instruments, Columbus, OH, USA). The grip test was repeated five times for each limb per testing session. Data from the dominant limb used for lever-pulling is reported.

### 2.6 Measures of systemic biomarkers

Tail vein blood was collected under anaesthesia (to avoid the stress of physical restraint needed otherwise (Wu et al., 2015)) at each time-point within the same time-window (0900–1100) to determine serum concentrations of BDNF, estradiol and corticosterone, and numbers of white and red blood cells. For the final *post-intervention* time-point, blood was collected 16 and 28 h after the final sleep disturbance or running wheel session to avoid any acute effects of these activities on blood analyte fluctuations (Cerqueira et al., 2019; Mullington et al., 2010).

Blood was collected, aliquots diluted, and white and red blood cells counted and calculated as number of cells per 1 mm^3^. The remaining blood was allowed to clot for ∼45 min in uncoated tubes before centrifuging at 12,000 revolutions/minute at 4 °C for 20 min. Immediately after centrifugation, serum was collected, aliquoted and stored at −80 °C until assayed. BDNF, estradiol and corticosterone levels were assessed separately using commercially available ELISAs. See Appendix A for details.

### 2.7 Statistical analysis

GraphPad Prism was used. Running distance was compared between both groups exposed to exercise: (1) exercise only (Ex) and (2) sleep disturbance plus exercise (SD+Ex). *Overall* running distance was compared with a two-tailed *t*-test and *over time* (pre-injury *vs.* post injury *vs.* post-intervention) with a repeated-measures mixed-effects model and cubic polynomial curve fitting. Pain behavioural measures and serum biomarkers were compared between *groups* (Ex, SD+Ex and sleep disturbance only [SD]) over *time* (pre-injury *vs.* post injury *vs.* post-intervention) using repeated-measures mixed-effects models. Tukey’s multiple comparisons or Fisher’s least significant difference post hoc tests were used to assess differences between *groups* over *time*. Spearman rank correlational tests were used to compare biomarker levels with behavioural results. P-values <0.05 were considered statistically significant. Data are expressed as mean ± SEM. Raw data, mean ± SEM, and repeated-measures mixed-effects model outcomes are presented in Appendices B and C. Post hoc outcomes are presented in figures.

## 3 Results

Cubic polynomial curve fitting indicated that Ex rats ran slightly more than SD+Ex rats from day 15 onwards (Fig. 1E), although the overall mean running distance was similar: Ex: 3.02 ± 0.3; SD+Ex: 2.45 ± 0.32 (mean ± SEM; p = 0.25). The average daily running distance was similar between these two *groups* (p = 0.09) over the four-week running period, with both running further as *time* progressed (p = 0.0004).

**Fig. 2:**
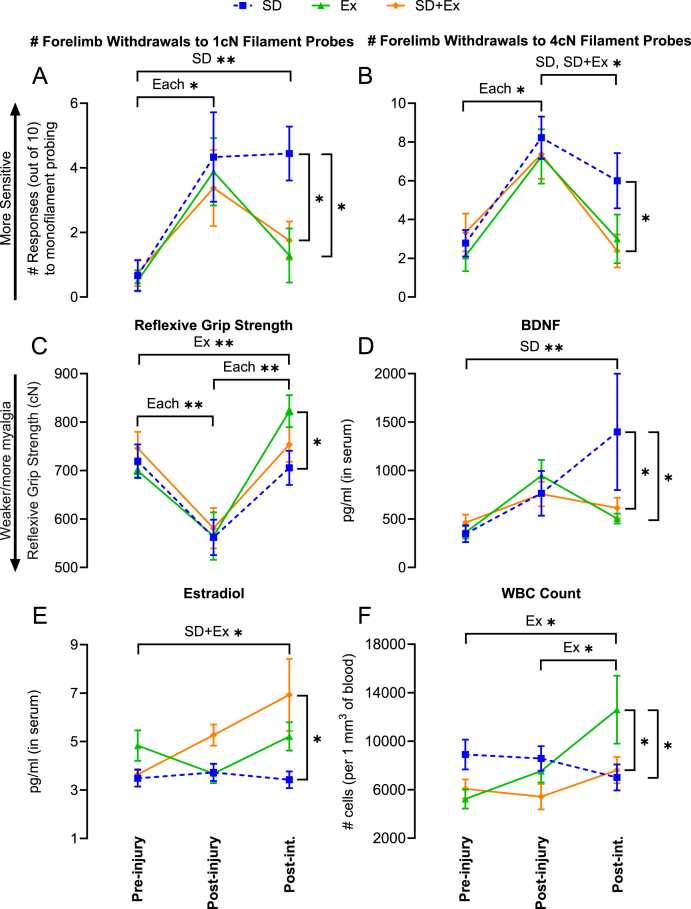
Behavioural and systemic biomarker outcomes. Effects of injury and post-injury sleep disturbance (SD), exercise (Ex) or both (SD+Ex) on: (A) forepaw mechanical sensitivity to 1 cN or 4 cN monofilament testing, (B) reflexive grip strength, (D–E) serum levels of BDNF and estradiol, and (F) whole blood levels of white blood cells. Post-int. = post-intervention. *p < 0.05, **p < 0.01, between groups as shown.

Forepaw mechanical sensitivity differed over *time* in response to 1 cN and 4 cN monofilament probing (p = 0.0002 and p < 0.0001, respectively). Sensitivity increased post-injury (i.e., after task performance) in all groups (Fig. 2A and B) and remained elevated after sleep disturbance alone, but decreased in response to exercise with and without sleep disturbance. Forepaw sensitivity to 8 cN monofilaments similarly lowered in response to exercise, but 0.4 cN monofilament probing was not different between groups or over time (see Appendix B for results).

Reflexive grip strength differed with *time* (p < 0.0001)—values decreased post-injury but increased post-intervention for each group (Fig. 2C). Reflexive grip strength values were higher post-intervention after exercise relative to sleep disturbance alone.

Serum biomarker (except for corticosterone) and blood cell count outcomes are presented in Fig. 2D-F. BDNF levels changed over *time* (p = 0.002) and there was a significant interaction between *group* and *time* (p = 0.04). BDNF remained similar between groups pre- and post-injury but increased post-intervention in response to sleep disturbance alone, but not in response to exercise with and without sleep disturbance (Fig. 2D). Levels also correlated positively and moderately with forepaw mechanical sensitivity in response to 1 cN and 4 cN monofilament probing (r = 0.39 and r = 0.44, respectively; p < 0.01). Estradiol levels were influenced by *group* (p = 0.01) and there was a significant interaction between *group* and *time* (p = 0.04). Post-injury and post-intervention levels of estradiol were higher (*versus* sleep disturbance alone) and increased over time in response to combined exercise and sleep disturbance but did not change in response to either sleep disturbance or exercise alone (Fig. 2E). Corticosterone levels did not differ between group or over time (Appendix C). With respect to blood cell counts, white blood cells (WBC) increased and were higher only in response to exercise (*group × time* interaction: p = 0.01; Fig. 2F), whereas red blood cells (RBC) were unchanged by time or any intervention (Appendix C).

## 4 Discussion

These findings support the hypothesis that poor sleep drives increased and persistent pain. Although prior studies have linked reduced/poor sleep with exacerbated pain perception, hyperalgesia and the risk of developing chronic pain (Finan et al., 2013), they are limited to short-term (<1 week) experimental or non-/poorly controlled observational studies. Our controlled pre-*versus* post-injury approach provides the first evidence of a potential causal relation between poor sleep and the transition from acute to persistent pain. Whether improved sleep reduces pain and the transition to chronicity via moderating systemic mediators involved in pain sensitization will be a critical point of future research and one with clear clinical implications.

The degree to which aerobic exercise offset the negative impact of poor sleep on post-injury sensitivity supports our hypothesis and highlights its potential use as an intervention to reduce/prevent persistent pain for those who have difficulty modifying their sleep (e.g., shift workers, chronic insomnia sufferers). It also opens the enticing, yet untested possibility that interventions aimed at sleep might improve pain, and that this effect might be amplified with the addition of exercise—which is a first-line therapy for musculoskeletal pain—as part of a multimodal treatment program. Confirmation of our finding and hypotheses could have enormous clinical impact as poor sleep affects one-in-three adults and coexists with chronic pain in up to 88% of cases (Smith et al., 2004; Finan et al., 2013).

Insight into the physiology underlying the dualling relationship between poor sleep and exercise can be drawn from the examined biomarkers. The increase in systemic BDNF with sleep disturbance (alone) likely reflects three factors. First, BDNF regulates sleep drive and architecture, and alterations in sleep have been shown to correlate with changes in BDNF levels (Rahmani et al., 2020). Second, central nervous system levels of BDNF (where ∼75% of BDNF is produced) highly correlate with systemic levels since BDNF readily crosses the blood–brain barrier (Li et al., 2020). Third, poor sleep can acutely enhance inflammation (Klyne et al., 2021), and inflammation is a known trigger for BDNF release (Nijs et al., 2015). This latter point is important because BDNF might be a mechanism by which inflammation contributes to the development and maintenance of central sensitization (Nijs et al., 2015). Our finding that higher BDNF correlated with greater mechanical sensitivity supports this view. Conversely, the opposite was observed in rats that exercised, regardless of sleep state. This matches past human data showing decreases in BDNF with long-term aerobic exercise (De et al., 2019). The abovementioned factors corroborate with the view that BDNF can sensitize and alter pain pathways at every level, from the peripheral nociceptor to the spinal cord and the brain, via its strong capacity to regulate synaptic plasticity (Nijs et al., 2015). Our data (Fig. 2A–D) support this and the emerging hypothesis that increases in BDNF can initiate and sustain central sensitization processes involved in the development of chronic pain (Nijs et al., 2015).

Potentially protective biomarkers induced by exercise were estradiol and WBCs (Fig. 2E and F). Estradiol has receptors throughout the entire body (including diversely through the brain) (Almey et al., 2015), has known analgesic effects (Wise et al., 2009; Athnaiel et al., 2023), and lower levels are associated with greater pain (Pang et al., 2023). Moreover, estrogenic activity is involved in regulating many pathways, including neuropeptide (e.g., BDNF) transcription in the brain (Balasubramanian et al., 2014). With respect to the increase in WBCs with exercise (only), we suspect that this is an adaptive response post-injury to stimulate clearance of injury-induced debris and tissue repair, as suggested previously (Lech et al., 2013). That WBCs did not increase post-injury with sleep disturbance, irrespective of exercise engagement, suggests that poor sleep might suppress certain immune responses required for optimal tissue healing and pain relief. A next step would be to explore the interrelationships between these factors and BDNF as pain evolves from acute to chronic.

Cortisol, which is the corticosterone equivalent in humans, responds to acute stressors such as those related to sleep deprivation (Leproult et al., 1997) and high intensity exercise (Hill et al., 2008). That corticosterone levels were unchanged by any intervention in the current study might, in part, reflect the *intended* “low” stress methods we applied for exercise (i.e., voluntary, not forced) and inducing poor sleep. There is also some data to suggest that cortisol and corticosterone respond differently to acute stressors (Ovejero et al., 2013). Clarification of this relationship is needed.

## 5 Conclusion

Our study provides first evidence of a role for poor sleep in the transition from acute to persistent pain, and for aerobic-based exercise to counter these effects. The data also highlight the potential importance of BDNF in these relationships. Confirmation of these findings in larger pre-clinical and human studies would have important clinical implications.

## CRediT authorship contribution statement

**David M. Klyne:** Conceptualization, Data curation, Formal analysis, Funding acquisition, Investigation, Methodology, Project administration, Writing – original draft, Writing – review & editing, Supervision. **Brendan A. Hilliard:** Data curation, Formal analysis, Investigation, Methodology, Supervision, Writing – review & editing. **Michele Y. Harris:** Data curation, Investigation, Writing – review & editing. **Mamta Amin:** Data curation, Investigation, Methodology, Writing – review & editing. **Michelle Hall:** Writing – review & editing. **Manuela Besomi:** Data curation, Writing – review & editing. **Sanam Mustafa:** Writing – review & editing. **Scott F. Farrell:** Writing – review & editing. **Oliver Rawashdeh:** Writing – review & editing. **Felicity Y. Han:** Writing – review & editing. **Paul W. Hodges:** Conceptualization, Writing – review & editing. **Nagat Frara:** Investigation. **Mary F. Barbe:** Conceptualization, Data curation, Formal analysis, Funding acquisition, Investigation, Methodology, Resources, Supervision, Writing – original draft, Writing – review & editing.

## Declaration of competing interest

No conflict of interest for all authors.

## Data Availability

Study data that are not included in the article are available in Appendices B and C.
