# Supplementary material for: Poor sleep versus exercise: A duel to decide whether pain resolves or persists after injury
Source: Brain Behav Immun Health. 2023 Dec 12;35:100714. doi: 10.1016/j.bbih.2023.100714 (PMC10727927; doi:10.1016/j.bbih.2023.100714)
Supplement: Multimedia component 1 [file mmc1.docx]

**APPENDIX A: SUPPORTING INFOMRATION FOR:**

Poor sleep versus exercise: A duel to decide whether pain resolves or persists after injury?

David M Klyne^1*^, Brendan A Hilliard^2^, Michele Y Harris^2^, Mamta Amin^2^, Michelle Hall^3^, Manuela Besomi^1^, Sanam Mustafa^4^, Scott F Farrell^5^, Oliver Rawashdeh^6^, Felicity Y Han^7^, Paul W Hodges^1^, Nagat Frara^2^, Mary F Barbe^2^

^1^The University of Queensland, NHMRC Centre of Clinical Research Excellence in Spinal Pain, Injury and Health, School of Health and Rehabilitation Sciences, Brisbane 4072, Australia; ^2^Aging + Cardiovascular Discovery Center, Lewis Katz School of Medicine, Temple University, Philadelphia, 19140, USA; ^3^Centre for Health, Exercise and Sports Medicine, School of Health Sciences, The University of Melbourne, Melbourne 3010, Australia; ^4^School of Biomedicine, The University of Adelaide, Adelaide 5005, Australia; ^5^The University of Queensland, RECOVER Injury Research Centre, Brisbane 4029, Australia; ^6^The University of Queensland, School of Biomedical Sciences, Brisbane 4072, Australia; ^7^The University of Queensland, Australian Institute for Bioengineering and Nanotechnology, Brisbane 4072, Australia.

^1^David M Klyne: E – [d.klyne@uq.edu.au](mailto:d.klyne@uq.edu.au), P – +61 7 3365 4569

**Extended Materials and Methods**

*1. Animals*

Ten to twelve week-old female Sprague-Dawley rats (*n*=30) brought from Charles River (Wilmington, MA) were allowed to acclimate to our facilities for at least 2 weeks prior to commencing study procedures. Rats were housed individually in standard rat cages (ventilated and with hardwood chip rodent bedding) in a central animal facility with a 12- hour light/12-hour dark cycle with free access to water. Rats were first acclimated to the animal facility for 1 week before a second week of handling (only) and the onset of food restriction – study rats were food restricted to 5% less (based on animal weight) than age-matched normal control rats with free-access-to-food; these latter rats were used for weight comparison purposes only. All rats were handled at least twice per week and provided cage enrichment toys daily, including chew bones, tunnels and paper twists (Diamond Twists, Teklad #7979C.CS, Envigo, South Easton, MA).

Rats were checked daily for health, feed and water by laboratory or institutional laboratory animal staff, under the supervision of the institutional veterinarian. All experiments were approved by the Institutional Animal Care and Use Committee and were compliant with NIH guidelines for the humane care and use of laboratory animals [1]. To reduce illness-related confounders, all animals were weighed weekly, examined at least twice weekly for indices of illness, and examined weekly for tumors (none were detected). Sentinel rats housed in the same housing room were examined for presence of illnesses as part of regular veterinary care (none were detected).

The decision to use only female rats was based on our injury model setup, which is currently tailored to the pulling strength of female rats. Inclusion of males would have reduced data quality and made the interpretation of findings difficult. Further, sex is an important factor in the modulation of pain [2, 3], and the inclusion of males would have introduced confound in the data that would have not been adequately controlled for due to our group sizes (n ≤10). To control for the effect of female sex hormones on pain behaviours, which vary at different stages of the estrous cycle (the equivalent of menstrual cycle in humans) [4], we assessed systemic estradiol levels. We found no group differences at time-points prior to the intervention phase (pre-injury, post-injury). Future studies that include both sexes are encouraged to consider these factors when interpreting pain data.

*2. Overall study design*

Following the induction of acute-onset overuse injury of the median nerve (see section 3), rats were randomly grouped and exposed for 4 weeks to either: (1) sleep disturbance (n=11), (2) voluntary whole body aerobic exercise (n=10 initially, n=9 by study end) or (3) both (n=9). Longitudinal measures of behavioural assays and systemic biomarkers were obtained at three times points: just prior to injury induction (pre-injury), immediately following the 4-week injury induction phase (post-injury), and immediately after the 4-week intervention/exposure phase (post-intervention).

*3. Acute-onset overuse injury by voluntary performance of a high repetition high force task*

Sixteen custom-designed operant behavioral chambers were used in which rats performed an operant reaching and lever pulling task, as previously described [5]. Following acclimation, all rats were trained for 4 weeks (15 min/day, 5 days/week) in these chambers to learn a reaching and lever-pulling task at high loads, ramping up from naïve loads slowly, at no specific reach rate, as previously described [5]. Rats then went on to perform a high-repetition high-force (HRHF) reaching and lever‐pulling task for 4 weeks in custom-designed behavioural apparatuses (Fig. 1, B-D). Briefly, animals reached through a shoulder height portal and isometrically pulled a force handle attached to a force transducer with a load cell (Futek Advanced Sensor Technology, Irvine, CA) located outside the chamber wall. The load cell was interfaced with custom Force-Lever software (version 1.03.02, Med Associates, St. Albans, VT). Auditory and light indicators cued the reaching rate. If reach and force criteria (see below) were met within a 5-second cueing period, a 45 mg food pellet was dispensed into a food trough as a reward. The reach and force criteria involved pulling the lever bar at 53% ±5% of the rats' maximum pulling force at a target reach rate of 4 reaches/min, for 2 hours/day, over four 30-minute intervals (separated by a 1.5-hour break), for 3 days/week (Monday, Wednesday and Friday). We have previously shown that this task induces sensorimotor declines, local and systemic inflammation [5, 6] that is consistent with that seen in humans with acute-onset upper extremity repetitive strain injuries [7, 8].

During the training and task period, rats were food restricted to weigh 5% less than age-matched normal control rats that had free access to food (these latter rats were used for weight comparison purposes only). This food restriction was necessary to motivate rats to work for a food reward. All rats were weighed twice per week, provided regular rat chow daily in addition to food reward pellets (banana [F0024] and chocolate grain-based [F0165] dustless precision pellets, Bio-Serv, Flemington, NJ, USA), and allowed to gain weight over the course of the experiment [9].

*4. Sleep disturbance*

Sleep disturbance involved frequent arousing of rats during the light-phase (12 h from 0600-1800, as rats are nocturnal) on four random days of the week to avoid circadian and sleep-pattern adaptations. This was achieved by replacing an old object with a new stimulatory object in their cage when the rat appeared to become drowsy. Stimulatory objects included plastic toys and tubes of different sizes, tube lids, marbles and nesting material. When necessary, additional arousal was achieved by disturbing the bedding/nest and/or introducing objects containing sucrose food reward pellets that can be sensed but not accessed by the animal. Cages were also exchanged twice a day (1200 and 1400) for fresh ones to provide additional stimulation. This method avoids the confounding impact of acute stress associated with current sleep restriction/disturbance methods and better reflects poor sleep in humans in the real world.

Traditional models of sleep disturbance and/or deprivation (partial, intermittent, or complete) do not relate well to the human experience. Three major issues underlie this problem. First, most models are stress-related, involving exposure to acute stressors (e.g., loud/unpredictable noises, foot shock, grid over water in which animals fall from their confined grid if they fall asleep) [10], which are known to substantially impact the immune system and pain [11-13]. Second, other models involve either pharmacological, genetic or invasive interventions, resulting in a host of side-effects unrelated to the normal effects of poor sleep [10]. Third, poor sleep is usually induced and studied acutely and/or only over a short period of time (i.e., less than a week) [10]. In humans, however, poor sleep is generally of lower intensity and endured over longer periods of time, especially in the context of pain [14, 15]. An additional but perhaps less critical issue is the fact that many, if not most, bouts of poor sleep occur and/or worsen in response to interacting with our environment past “normal” bedtime hours. Most current animal models do not account for these important “real-world” factors. Our method used here addresses these issues.

*5. Voluntary exercise*

Exercise was in the form of voluntary running via free access to a built-in running wheel (Panlab Harvard Apparatus, Holliston, MA, USA) from within their cage for 12 hours during the dark-phase (1800-600), 5 days a week. We have had previous success with this method of “voluntary” exercise [16, 17] which avoids problems associated with forced exercise [9]. Exercise above a certain threshold can have negative effects including tissue damage and enhanced pain [18-20]. Exercise on a voluntary basis helps to negate these issues, as well as those related to the psychological stress induced by forced exercise [21, 22]. Daily wheel revolutions were recorded digitally using an automated rotameter (LE3806 multicounter; Harvard Apparatus) and exported using SeDaCom software – no differences in total running distance were observed between groups that were allowed access to a running wheel. Running wheel access was not permitted between 600-1800 (light-phase) to avoid the stimulatory effects of the task which could alter normal sleep patterns. Rats were returned to their usual cage (i.e., without a wheel) during the light-phase. “Non-exercise” rats were housed in their usual cage without wheels.

*6. Longitudinal measures*

Measures of forepaw mechanical sensitivity, reflexive grip strength (as a measure of muscle myalgia), and systemic biomarkers were collected at three time-points: (1) pre-injury, (2) post-injury, and (3) post-intervention.

*6.1 Mechanical sensitivity*

Forepaw sensitivity to mechanical stimulation was assessed at both forepaws using von Frey monofilaments (North Coast Medical, Inc. CA) as previously described and performed [23]. Rats were placed in an elevated apparatus consisting of a wire-rung floor (spaced 8 mm apart), with each rat separated by acrylic dividers, and allowed to acclimate for 5 to 10 minutes. When alert, settled and weight-bearing equally on all four paws, von Frey monofilaments (0.4 cN, 1 cN, 4 cN, and 8 cN) were applied to the forepaws in the ascending order of force. Each filament was applied 10 times, in a pseudorandom order, to the midplantar surface of each forepaw, and held in place for 5 seconds. The number of withdrawal responses was recorded to give a score out of a maximum of 10 for each von Frey monofilament at each forepaw, with higher scores indicative of greater sensitivity. For analysis purposes, scores for the dominant “reaching” limb were considered. The person who carried out these assays was an experienced tester and blinded to group assignment. This method measures cutaneous sensitivity (termed hyperalgesia or allodynia in humans) and is thus most useful for mimicking clinical conditions with enhanced cutaneous sensitivity, such as occurs with neuropathic pain, nociplastic pain and inflammation [24-26].

*6.2 Reflexive grip strength*

Reflexive grip strength was tested using a rat grip strength meter (1027SR-D58, Columbus Instruments, Columbus, Ohio). The test was repeated 5 times/limb at each time-point. Maximum grip strength per trial is reported. As for mechanical sensitivity, data from the dominant limb used for reaching were anlysed.

*6.3 Systemic biomarkers*

Tail vein blood was collected under anaesthesia (to avoid stressing the animal) at each respective time-point within the same time-window (0900-1100) for determination of systemic concentrations of brain-derived neurotrophic factor (BDNF), estradiol and corticosterone, and numbers of white and red blood cells. Blood was collected into uncoated tubes, allowed to clot for ~45 min, and then centrifuged at 12000 revolutions per minute at 4 °C for 20 minutes. Serum (the supernatant) was collected and immediately aliquoted into 200 μl microcentrifuge tubes and stored at -80 °C until assayed.

BDNF, estradiol and corticosterone levels were assessed using separate commercially available [enzyme-linked immunosorbent assays](https://www.sciencedirect.com/topics/immunology-and-microbiology/enzyme-linked-immunosorbent-assay): BDNF (Rat Quantikine ELISA Total BDNF, DBNT00, analytical sensitivity of 1.35 pg/ml, R&D Systems, Minneapolis, MN, USA); mouse/rat estradiol (ES180S-100, analytical sensitivity of 3 pg/ml, CalBiotech Inc., El Cajon, CA, USA); and mouse/rat corticosterone (55-CORMS-E01, analytical sensitivity of 6.1 ng/ml, Alpco, Salem, NH, USA). Absorbance was measured according to the manufacturer’s directions using the Paradigm (Beckman Coulter, Inc., CA) microplate reader. Values below the sensitivity of the test were allocated a zero score.

White blood cells were counted by diluting 5 ml of blood in 95 ml of Turk’s solution: 0.1% methylene blue in 3% acetic acid. Leukocyte nuclei were counted and the number of cells in 1 mm^3^ of undiluted blood was calculated. Red blood cells were counted by diluting 5 ml of blood in 995 ml of normal saline. The diluted blood was mixed thoroughly, and 10 ml applied to a hemocytometer or counting chamber. Red blood cells were counted and the number of cells in 1 mm^3^ of undiluted blood was calculated.

*7. Statistical analysis*

GraphPad PRISM version 10 for Macs was used for statistical analyses. The sample size for this study was based on our prior work showing a minimum of 5 rats per group were needed for the various assays [27, 28]. Six to eleven rats per group were included for each analysis. A two-tailed t-test was used to compare overall average running distance between the *exercise only* (Ex) and *sleep deprivation plus exercise* (SD+Ex) groups across the four intervention weeks. A repeated-measures mixed-effects model (Restricted Maximum Likelihood, REML) and cubic polynomial curve fitting were used to determine if running distance differed between these two *Groups* (Ex *vs.* SD+Ex) over *Time* (daily over the 4-week intervention period). Behavioural outcomes and systemic biomarkers were compared between *Groups* (SD *vs.* Ex *vs.* SD+Ex) over *Time* (pre-injury *vs.* post-injury *vs.* post-intervention) using repeated measures mixed-effects models (REML). Tukey’s multiple comparisons or Fisher’s least significant difference post hoc tests were used to assess differences between groups and/or time-points. P-values <0.050 were considered statistically significant and all data are expressed as mean ±SEM. Post hoc outcomes are presented in figures.

**Data availability**

Raw data, mean ± SEM, and repeated-measures mixed-effects model outcomes are shown in Appendices B and C.

**References**

1. Grundy, D., *Principles and standards for reporting animal experiments in The Journal of Physiology and Experimental Physiology.* J Physiol, 2015. **593**(12): p. 2547-9.

2. Bartley, E.J. and R.B. Fillingim, *Sex differences in pain: a brief review of clinical and experimental findings.* Br J Anaesth, 2013. **111**(1): p. 52-8.

3. Popescu, A., L. LeResche, E.L. Truelove, and M.T. Drangsholt, *Gender differences in pain modulation by diffuse noxious inhibitory controls: a systematic review.* Pain, 2010. **150**(2): p. 309-318.

4. Vinogradova, E.P., D.A. Zhukov, and A.S. Batuev, *The effects of stages of the estrous cycle on pain thresholds in female white rats.* Neurosci Behav Physiol, 2003. **33**(3): p. 269-72.

5. Barbe, M.F., et al., *The interaction of force and repetition on musculoskeletal and neural tissue responses and sensorimotor behavior in a rat model of work-related musculoskeletal disorders.* BMC Musculoskelet Disord, 2013. **14**: p. 303.

6. Barbe, M.F., et al., *Blocking CTGF/CCN2 reduces established skeletal muscle fibrosis in a rat model of overuse injury.* FASEB J, 2020. **34**(5): p. 6554-6569.

7. Carp, S.J., et al., *Inflammatory biomarkers increase with severity of upper-extremity overuse disorders.* Clin Sci (Lond), 2007. **112**(5): p. 305-14.

8. Gold, J.E., et al., *Systematic review of biochemical biomarkers for neck and upper-extremity musculoskeletal disorders.* Scand J Work Environ Health, 2016. **42**(2): p. 103-24.

9. Smith, T.T.G., et al., *Forced treadmill running reduces systemic inflammation yet worsens upper limb discomfort in a rat model of work-related musculoskeletal disorders.* BMC Musculoskelet Disord, 2020. **21**(1): p. 57.

10. Toth, L.A. and P. Bhargava, *Animal models of sleep disorders.* Comp Med, 2013. **63**(2): p. 91-104.

11. Chapman, C.R., R.P. Tuckett, and C.W. Song, *Pain and stress in a systems perspective: reciprocal neural, endocrine, and immune interactions.* J Pain, 2008. **9**(2): p. 122-45.

12. Silverman, M.N. and E.M. Sternberg, *Glucocorticoid regulation of inflammation and its functional correlates: from HPA axis to glucocorticoid receptor dysfunction.* Ann N Y Acad Sci, 2012. **1261**: p. 55-63.

13. Slavich, G.M. and M.R. Irwin, *From stress to inflammation and major depressive disorder: a social signal transduction theory of depression.* Psychol Bull, 2014. **140**(3): p. 774-815.

14. Finan, P.H., B.R. Goodin, and M.T. Smith, *The Association of Sleep and Pain: An Update and a Path Forward.* Journal of Pain, 2013. **14**(12): p. 1539-1552.

15. Medic, G., M. Wille, and M.E. Hemels, *Short- and long-term health consequences of sleep disruption.* Nat Sci Sleep, 2017. **9**: p. 151-161.

16. James, G., et al., *ISSLS Prize in Basic science 2019: Physical activity attenuates fibrotic alterations to the multifidus muscle associated with intervertebral disc degeneration.* Eur Spine J, 2019. **28**(5): p. 893-904.

17. James, G., M. Millecamps, L.S. Stone, and P.W. Hodges, *Dysregulation of the Inflammatory Mediators in the Multifidus Muscle After Spontaneous Intervertebral Disc Degeneration SPARC-null Mice is Ameliorated by Physical Activity.* Spine (Phila Pa 1976), 2018. **43**(20): p. E1184-E1194.

18. Sluka, K.A., J. Danielson, L. Rasmussen, and L.F. Dasilva, *Exercise-Induced Pain Requires NMDA Receptor Activation in the Medullary Raphe Nuclei.* Medicine and Science in Sports and Exercise, 2012. **44**(3): p. 420-427.

19. Staud, R., M.E. Robinson, and D.D. Price, *Isometric exercise has opposite effects on central pain mechanisms in fibromyalgia patients compared to normal controls.* Pain, 2005. **118**(1-2): p. 176-184.

20. Lima, L.V., T.S.S. Abner, and K.A. Sluka, *Does exercise increase or decrease pain? Central mechanisms underlying these two phenomena.* Journal of Physiology-London, 2017. **595**(13): p. 4141-4150.

21. Commons, K.G., A.B. Cholanians, J.A. Babb, and D.G. Ehlinger, *The Rodent Forced Swim Test Measures Stress-Coping Strategy, Not Depression-like Behavior.* Acs Chemical Neuroscience, 2017. **8**(5): p. 955-960.

22. Moraska, A., et al., *Treadmill running produces both positive and negative physiological adaptations in Sprague-Dawley rats.* American Journal of Physiology-Regulatory Integrative and Comparative Physiology, 2000. **279**(4): p. R1321-R1329.

23. Clark, B.D., et al., *Performance of a high-repetition, high-force task induces carpal tunnel syndrome in rats.* J Orthop Sports Phys Ther, 2004. **34**(5): p. 244-53.

24. Gregory, N.S., et al., *An overview of animal models of pain: disease models and outcome measures.* J Pain, 2013. **14**(11): p. 1255-69.

25. Lindblom, U. and R.T. Verrillo, *Sensory functions in chronic neuralgia.* J Neurol Neurosurg Psychiatry, 1979. **42**(5): p. 422-35.

26. Baumgartner, U., et al., *Neurogenic hyperalgesia versus painful hypoalgesia: two distinct mechanisms of neuropathic pain.* Pain, 2002. **96**(1-2): p. 141-51.

27. Barbe, M.F., et al., *Blocking CCN2 Reduces Progression of Sensorimotor Declines and Fibrosis in a Rat Model of Chronic Repetitive Overuse.* J Orthop Res, 2019. **37**(9): p. 2004-2018.

28. Bove, G.M., et al., *Manual therapy prevents onset of nociceptor activity, sensorimotor dysfunction, and neural fibrosis induced by a volitional repetitive task.* Pain, 2019. **160**(3): p. 632-644.
